# Using Nanoinformatics Methods for Automatically Identifying Relevant Nanotoxicology Entities from the Literature

**DOI:** 10.1155/2013/410294

**Published:** 2012-12-27

**Authors:** Miguel García-Remesal, Alejandro García-Ruiz, David Pérez-Rey, Diana de la Iglesia, Víctor Maojo

**Affiliations:** ^1^Departamento de Inteligencia Artificial, Facultad de Informática, Universidad Politécnica de Madrid, Boadilla del Monte, 28660 Madrid, Spain; ^2^Biomedical Informatics Group, Facultad de Informática, Universidad Politécnica de Madrid, Boadilla del Monte, 28660 Madrid, Spain

## Abstract

Nanoinformatics is an emerging research field that uses informatics techniques to collect, process, store, and retrieve data, information, and knowledge on nanoparticles, nanomaterials, and nanodevices and their potential applications in health care. In this paper, we have focused on the solutions that nanoinformatics can provide to facilitate nanotoxicology research. For this, we have taken a computational approach to automatically recognize and extract nanotoxicology-related entities from the scientific literature. The desired entities belong to four different categories: nanoparticles, routes of exposure, toxic effects, and targets. The entity recognizer was trained using a corpus that we specifically created for this purpose and was validated by two nanomedicine/nanotoxicology experts. We evaluated the performance of our entity recognizer using 10-fold cross-validation. The precisions range from 87.6% (targets) to 93.0% (routes of exposure), while recall values range from 82.6% (routes of exposure) to 87.4% (toxic effects). These results prove the feasibility of using computational approaches to reliably perform different named entity recognition (NER)-dependent tasks, such as for instance augmented reading or semantic searches. This research is a “proof of concept” that can be expanded to stimulate further developments that could assist researchers in managing data, information, and knowledge at the nanolevel, thus accelerating research in nanomedicine.

## 1. Introduction

Nanoinformatics is a nascent research field at the intersection of several disciplines, including informatics (information technologies and computer science), nanotechnology, medicine, biology, chemistry, and physics [1]. Nanoinformatics refers to the practical application of information technologies to gather, store, retrieve, and process information, data, and knowledge on the physicochemical characteristics of nanoparticles, nanomaterials, and nanodevices and their potential applications, especially in the biomedical field [1]. 

Applications of nanoinformatics include, for instance, nanoparticle characterization and design, modeling and simulation, data integration and exchange, linking nanoparticles information to clinical data, semantic annotation and retrieval, domain ontologies, terminologies and standards, and data and text mining for nanomedical research [2]. In this context, we can recall and emphasize the role that bioinformatics—a related informatics discipline—played in accelerating the Human Genome Project. One can conjecture that nanoinformatics might play the same role for nanotechnology and nanomedicine that bioinformatics and medical informatics have played in biology and medicine. We have already begun to define the role that nanoinformatics could play for nanomedicine, as reported elsewhere [3, 4]. 

In our recent work in this field, we have focused on the challenges, opportunities, and solutions that nanoinformatics can provide to a critical subfield of nanomedicine: nanotoxicology. This discipline aims to determine whether and to what extent the unique properties of nanoparticles (that arise due to questions such as quantum size effects and/or their large surface-to-volume ratio) may present potential or real threats to humans, the environment, or to other species.

Publications have recently highlighted how nanoparticles enable a wide range of applications for clinical and therapeutic purposes. Bolhassani and colleagues [5] discuss the use of different types of nanoparticles, such as dendrimers, polymeric nanoparticles, metallic and magnetic nanoparticles, and quantum dots as effective vaccine adjuvants for infectious diseases and cancer therapy. Kosuge and colleagues [6] report the use of FeCo/graphitic-carbon nanocrystals (FeCo/GCNs) to enhance cellular fluorescence and magnetic resonance imaging of vascular inflammation due to their accumulation in vascular macrophages in vivo. Similarly, Thakor and colleagues [7] describe the use of polyethylene glycosylated Raman-active gold nanoparticles (PEG-R-AuNPs) in different clinical trials targeting dysplastic bowel lesions during colonoscopy. More extensive reviews can be found in [8, 9].

Despite these advances, the use of nanoparticles may involve serious risks for both patients and environment due to potential secondary toxic effects, also reported in the literature [10–14]. Therefore, it is essential for clinicians and researchers using nanoparticles for therapeutic purposes to be able to access relevant nanotoxicology information in an integrated and intuitive manner. Similarly, regulatory and environmental researchers need data and information integration in performing risk assessments or environmental forecasts as the result of manufacturing, use, degradation, disposal, and recycling of these materials. Taking advantage of nanoinformatics methods—most specifically text mining and natural language processing techniques applied to toxicological issues—should contribute to automatically identifying, organizing and making available specific nanotoxicity information reported in the literature to researchers and physicians.

Based on related research, we have carried out in the Biomedical Informatics field (BMI) [15–18], we present in this paper a nanoinformatics approach based on named entity recognition (NER) techniques for automatically extracting nanotoxicology-related entities from the literature. This, to our knowledge, is the first reported effort to automatically identify and extract relevant entities from scientific papers relevant to nanotoxicology. The extracted entities include, for instance, names of nanoparticles, nanomaterials, and nanodevices, types of toxicity/damage—for example, cell death or lung inflammation—and potential routes of exposure to toxic agents—for instance, inhalation or dermal contact. Once this information is retrieved and gathered, it can be used for a wide variety of applications.

This paper is organized as follows. In the background Section 2, we provide a survey of existing NER-focused methods and tools, most of them developed in the context of bioinformatics and medical informatics research. In the methods Section 3, we describe the building of the nanotoxicology training corpus, the training and construction of the automated entity recognizer, and the design of the evaluation experiment. Next, we present and discuss the results of the evaluation. Finally, we present the conclusions.

## 2. Background

Over the past few years, named Entity recognition (NER) methods and techniques have been widely used in medical informatics and bioinformatics research to automatically identify and extract different types of named entities (NEs) such as gene and/or protein names [19–23], medications and dosages [24], primary diseases and comorbidities [24], or raw sequences of nucleic acids and proteins [16, 20, 25, 26].

According to Park and Kim [27], there are four main approaches to performing NER from textual sources: (1) dictionary-based approaches, (2) rule-based approaches, (3) machine-learning approaches and (4) hybrid approaches. Dictionary-based approaches, try to identify entity names belonging to domain-specific controlled vocabularies, taxonomies and/or ontologies directly from the literature. There are different techniques for matching entities mentioned in the text to dictionary entries. These include, simple pattern-matching [28–30] or statistical techniques [31] to compare sequences of tokens from the text to dictionary entries, advanced symbolic natural language processing and computational linguistic techniques such as those used in the National Library of Medicine's MetaMap program [32, 33], and innovative hybrid approaches such as the one described in [34]. This encodes both biomedical texts and dictionary entries into sequences of nucleotide symbols—i.e., A, C, G, and T. Once the dictionary entries and the textual documents have been converted into sequences, the authors use BLAST [35]—the most ubiquitous tool for DNA and protein sequence matching—to automatically identify the entity instances in the text. Although dictionary-based approaches are relatively simple to design and implement if the appropriate dictionary is available, they have several limitations. These include false positive and false negative recognition issues arising from ambiguous names and from synonym and spelling variants, respectively.

Rule-based approaches address some of the limitations of dictionary-based approaches by dealing with morphological variants not covered by the latter approaches [27]. Rule-based methods resort to handcrafted patterns and rules to deal with the different types of morphological variants. Some examples of rule-based approaches include [36–42]. The main disadvantage of rule-based approaches is the difficulty to adapt or reuse them for different domains.

In contrast to rule-based methods—that use handcrafted rules and patterns—machine learning approaches are aimed at “learning” predictive models that can be used to automatically detect the occurrence of NEs in the text. Examples of machine learning methods and techniques used for NER include conditional random fields [21, 43–46], hidden markov models [47–49], support vector machines [45], and context-aware rule-based classifiers [33, 50]. To automatically generate the desired predictive models, nearly all machine learning-based approaches require a set of documents to train the model. This training set is a body of text documents (often just single passages) that has been manually analyzed and annotated by domain experts to identify different entities occurring in them. Examples of widely used corpora in the biological domain include GENIA [51]—an annotated body of literature related to the MeSH terms “human”, “blood cells”, and “transcription factors”— the BioCreAtIvE body for Task 1A [52]—text passages annotated with names of genes and related entities—, or Linnaeus [53]—aimed at recognizing and identifying species names in the biomedical literature. Similar corpora—although considerably smaller in size—have been developed for the medical domain. These include, for instance, the corpora used in the I2B2 medication extraction challenge [24, 54] and the I2B2 Obesity NLP Challenge [55], or a recently developed corpus aimed at the automated discovery of anaphoric relations in clinical narrative [56]. 

Finally, hybrid approaches combine two or more of the previously described techniques to achieve better performance, since each of the described approaches have its own advantages and disadvantages. Examples of hybrid systems approach include [45], which combines two machine learning algorithms (conditional random fields and support vector machines) with several rule-based engines, the approaches described in [16, 33], that rely on rule-based systems and lookup lists, or the hybrid method reported in [20] that describes a system combining a preprocessing dictionary and a rule-based filter with several independently trained support vector machines. 

After reviewing the results of recent NER-related challenges [24, 55], we decided to adopt a machine learning approach based on conditional random fields (CRFs) to build our nanotoxicology-related named entity recognizer. We made this decision since CRF-based biomedical NER systems are fast, effective, accurate, and perform relatively well even if trained with small training sets [21, 24, 55]. The latter issue is critical for the purpose reported in this paper, since to our knowledge there are no any available corpora for the nanotoxicological domain. Therefore, we had to build our own nanotoxicology corpus from scratch, which is a difficult and time-consuming task.

In the next section, we describe (1) the methods we used to build the corpus for training and evaluating the recognizer, (2) the CRF training process, and (3) the metrics we used to evaluate the performance of the nanotoxicology-related named entity recognizer.

## 3. Methods

The proposed NER system is designed to recognize instances of entities belonging to four different categories: NANO, EXPO, TOXIC, and TARGET. Entities belonging to the NANO category represent nanoparticles, nanodevices, or nanomaterials, such as for instance, *“polyamidoamine dendrimers”* or *“buckminsterfullerene”*. Similarly, EXPO-labeled instances describe different routes of human, animal, or environmental exposure to nanoparticles, such as *“inhalation”*, *“dermal contact”* or *“pulverization”*. On the other hand, TOXIC-labeled terms represent toxicological hazards of nanoparticles such as *“detachment” *or *“death”*, while TARGET-labeled terms refer to the actual targets of the hazards such as *“cell” *or *“kidney”*. 

We trained a CRF model using an annotated corpus containing 300 sentences selected from the available literature. Further details on the creation of the annotated corpus, the training of the CRF model and the evaluation protocol follow.

### 3.1. Building the Annotated Corpus

To build the corpus, we submitted the query “nanoparticles/toxicity(MeSH major topic)” to PubMed, obtaining 654 results at the time of writing. We manually analyzed the resulting set of abstracts to choose 300 sentences containing relevant entities. Members of our research group manually annotated the selected sentences. Both the selection of the 300 sentences and the annotation process were validated by two experts in nanomedicine and nanotoxicology.

**Figure 1:**
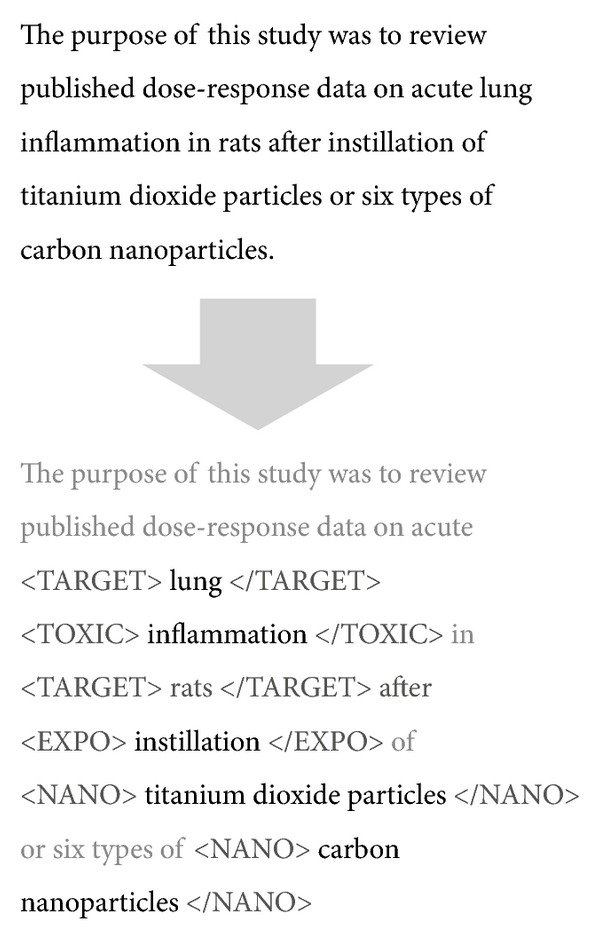
Sample annotated sentence belonging to the current “gold standard”, containing 6 different mentions of entities belonging to different categories.

The outcome of the labeling process was an annotated set of 300 sentences.  Figure 1 shows a sample annotated sentence containing instances for all the target categories. As depicted in the figure, each entity is enclosed between an opening and ending tag that denotes the category to which it belongs. For this sample sentence, we have two different instances belonging to the NANO category: *“titanium dioxide particles”* and *“carbon nanoparticles”*, one to EXPO: *“instillation”*, two to TARGET: *“lung” *and *“rats*”, and one to TOXIC: *“inflammation”*.

Table 1 summarizes the number of entities belonging to each category that were identified in the 300 selected phrases and labeled as such by the annotators. As entities may be composed of 2 or more words (tokens), such as for instance *“titanium dioxide particles”*, the table also reports the total number of tokens belonging to each category. Thus, the mention *“titanium dioxide particles”*, belonging to the NANO category at entity-level would be counted as 3 different mentions of the NANO category at the token-level. We made this distinction to evaluate the performance of the system both at entity-level (exact matching) and at token level (partial or inexact matching). Further detail is given in the section *Evaluation Metrics*.

### 3.2. Training the CRF Model

We trained a CRF model on the 300 annotated sentences to automatically identify instances of entities belonging to the four target categories. To train the CRF, we used the Java Application Programming Interface (API) provided by ABNER [21]. The latter is an open-source named entity recognizer designed to identify protein names and gene products. The model was trained using the default set of features provided by ABNER that includes orthographic, morphological, and contextual features. The latter are mostly based on regular expressions and n-gram features. We also performed minor modifications on the default tokenizer supplied with ABNER to properly identify chemical formulas.

### 3.3. Evaluation Metrics

We assessed the performance of the CRF-based NER system by calculating the precision, recall, and *F*-measure values for each type of entity—that is, NANO, EXPO, TOXIC, and TARGET. These metrics were computed both at entity and token levels [54]. Entity-level metrics measure the ability of the system to successfully recognize the full text of multiword entities labeled as such in the gold standard—i.e., the training set of manual annotations in the corpus. Conversely, token-level metrics are targeted at evaluating the performance of the system when labeling individual words. For instance, let us suppose that the annotation provided by our system for the sentence *“In this study, metallic nickel nanoparticles caused higher…”* is *“In this study, metallic <NANO>nickel nanoparticles</NANO> caused higher…”*, and that the provided annotation for this sample sentence in the gold standard is *“In this study, <NANO>metallic nickel nanoparticles</NANO> caused higher…”*. Therefore, for this example, the system would fail to provide a correct entity-level annotation for the NANO-labeled entity “*metallic nickel nanoparticles*”, since the system only achieved a partial match. However, this annotation would lead to an increase in recall for the NANO category at the token level, since the system successfully recognized 2 tokens (out of 3) in the phrase “*metallic nickel nanoparticles*” as belonging to the NANO category. We used formulas (1) to compute entity-level and token-level precision, recall, and *F*-measure:

(1) $$\begin{array}{c} \text{Entity-level}\;\text{Precision}\;\;\left(\mathrm{EP}\right)=\frac{\#\text{correctly}\;\;\text{returned}\;\;\text{entities}\;\;\text{by}\;\;\text{system}}{\#\text{entities}\;\;\text{returned}\;\;\text{by}\;\;\text{system}},\\ \text{Entity-level}\;\text{Recall}\;\;\left(\text{ER}\right)=\frac{\#\text{correctly}\;\;\text{returned}\;\;\text{entities}\;\;\text{by}\;\;\text{system}}{\#\text{entities}\;\;\text{in}\;\;\text{gold}\;\;\text{standard}},\\ \text{Entity-level}\;F\text{-measure}\;\;\left(\text{EF}\right)=\frac{2\cdot \mathrm{EP}\cdot \text{ER}}{\mathrm{EP}+\text{ER}},\\ \text{Token-level}\;\text{Precision}\;\;\left(\text{TP}\right)=\frac{\#\text{correctly}\;\text{returned}\;\text{tokens}\;\text{from}\;\text{each}\;\text{entity}\;\text{in}\;\text{system}\;\text{output}}{\#\;\;\text{tokens}\;\;\text{in}\;\;\text{system}\;\;\text{output}},\\ \text{Token-level}\;\text{Recall}\;\;\left(\text{TR}\right)=\frac{\#\text{correctly}\;\text{returned}\;\text{tokens}\;\text{from}\;\text{each}\;\text{entity}\;\text{in}\;\text{system}\;\text{output}}{\#\;\;\text{tokens}\;\;\text{in}\;\;\text{gold}\;\;\text{standard}},\\ \text{Token-level}\;F\text{-measure}\;\left(\text{EF}\right)=\frac{2\cdot \text{TP}\cdot \text{TR}}{\text{TP}+\text{TR}}. \end{array}$$

Although the size of the set of annotated sentences—in terms of number of sentences, entities, and tokens—is reasonable and could be divided into a training and test set to evaluate the system's performance, we instead chose to use 10-fold cross-validation to avoid overfitting. In the next section, we report the results of the evaluation activity.

## 4. Results and Discussion

Table 2 summarizes the results of the evaluation of the CRF-based entity identifier against the manually annotated gold standard using 10-fold cross-validation. The table shows the precision, recall, and *F*-measure for each target category (NANO, EXPO, TOXIC, and TARGET) both at entity and token level.

As shown in Table 2, our CRF-based entity recognizer yields entity-level precision values that range from 87.60% (TARGET) to 93.00% (EXPO). Similarly, entity-level recall values range from 82.60% (EXPO) to 87.40% (TOXIC). Performance of the recognizer at the token level, include precision values ranging from 90.06% (TARGET) to 98.10% (EXPO), while recall values range from 85.50% (EXPO) to 94.30% (NANO). These results show that the CRF-based approach performs particularly well at recognizing nanotoxicology entities—with entity-level *F*-measure values close to 90% for all categories—even when trained with such a reduced set of sentences. Moreover, the CRF-based approach seems to perform better at recognizing nanotoxicology entities than at identifying entities belonging to the biomedical domain, as reported for protein and gene names (precision = 65.90%, recall = 74.50%) [21], or medication information (precision = 90.37%, recall = 66.12%) [44]. The targeted entities are, of course, quite different, so direct comparisons should be treated with caution. 

To ensure a fair evaluation, we compared the adopted CRF-based approach to a hybrid approach used as baseline. This hybrid method combines a dictionary-based approach with a term selection scheme based on TF/IDF (term frequency/inverse document frequency) weights [57]. The latter are widely accepted statistics that measure the importance of a term in the context of a textual collection or corpus. To evaluate the hybrid method used as baseline, we proceeded as follows. First, we built a dictionary containing all terms occurring in the corpus we created, composed of 300 sentences. This dictionary contained all tokens—excluding stop words—and n-grams of sizes ranging between 2 and 6 occurring in the corpus. N-grams are groups of tokens that appear consecutively in the text. For instance, for the sentence *“Gold nanoparticles have the potential to …”* we would have the following n-grams of size 2: *“Gold nanoparticles”, “nanoparticles have”, “have the”, “the potential”, “potential to”.* Examples of n-grams of size 3 include *“Gold nanoparticles have”* or *“nanoparticles have the”*. We chose using n-grams in addition to single-word tokens since many concepts belonging to different ontologies are multiword concepts. Next, for each term *T* in the vocabulary, we calculated its TF/IDF score for the document containing the maximum number of occurrences of the term *T*. After that, all terms in the vocabulary were sorted in descending order of the TF/IDF score. We discarded all terms having a TF/IDF score smaller than *0.1*. Finally, we compared the remaining terms in the vocabulary to terms belonging to two different ontologies: the Foundational Model of Anatomy [58]—to detect anatomical locations that might be potential targets of nanoparticles—and the Nanoparticle Ontology [59]—to identify names of nanoparticles. If a term from the vocabulary matched a term from any of the ontologies, then it was marked as belonging to the NANO category—if the matched term belonged to the Nanoparticle Ontology—or to the TARGET category—if the matched term belonged to the Foundational Model of Anatomy. Note that, we did not focus on identifying toxic effects of nanoparticles and modes of exposition since there are no currently available ontologies or controlled vocabularies addressing such topics, and thus it is not possible using a vocabulary-based approach.  Table 3 shows the results of the evaluation for the method used as baseline.

As shown in Table 3, the baseline approach yields precisions of 100% and 75% for the NANO and TARGET categories respectively. These figures are reasonable, since most terms matching concepts belonging to the Nanoparticle Ontology refer to names of nanoparticles with high probability. This is not the case, however, for terms matching concepts from the Foundational Model of Anatomy, since anatomical locations may be mentioned together with nanoparticle names and there not might exist any toxicity relationships between them. Regarding the recall values yielded by the baseline method, it must be noted that these values are much smaller than those yielded by the CRF-based approach. These values are also reasonable, since the Nanoparticle Ontology was initially designed to provide a conceptualization of the domain of cancer nanotechnology research, while the documents in the corpus are targeted at different diseases. Similarly, the Foundational Model of Anatomy alone is not suitable for detecting potential targets of nanoparticles, since in addition to anatomical locations, potential targets of nanoparticles may also include animals and the environment. 

These results suggest that the CRF-based approach is suitable for performing NER-dependent tasks, especially when other approaches such as the vocabulary-based one cannot be performed due to the lack of a well-established controlled vocabularies or ontologies. 

**Figure 2:**
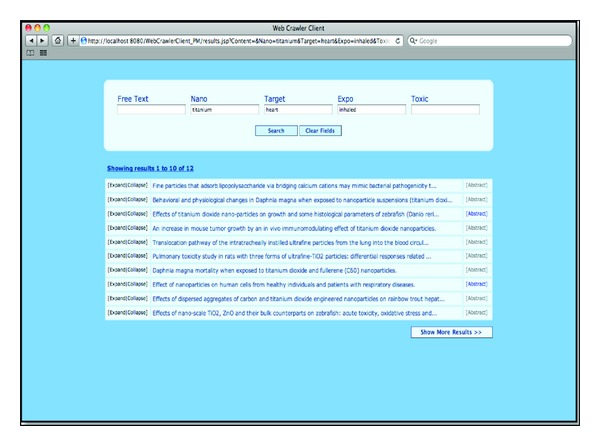
Screenshot of the prototype of the “nanotoxicity searcher”.

Examples of NER-dependant tasks that can be carried out using our nanotoxicology recognizer include, for instance, finding relationships between the detected entities, or indexing scientific papers with the different entities appearing in them. In fact, the latter task is a significant research topic in biomedical informatics research, since many different systems for automatically indexing and searching the biomedical literature have been developed over the last few years. Examples include Pharmspresso [60], an information retrieval and extraction system for pharmacogenomic-related literature that follows a dictionary-based approach to identify instances of genes, drugs, polymorphisms and diseases, or PubDNA Finder [17], an online repository that we developed to link PubMed Central manuscripts to the sequences of nucleic acids appearing in them, following a hybrid approach that combines a rule-based system and lookup lists. We have already begun working in this direction with the development of a prototype of the “nanotoxicity searcher”. The latter is an intelligent search engine that provides users with a web interface to search for PubMed-indexed papers that were automatically annotated with specific mentions of relevant nanotoxicology entities using the methods described in this paper.  Figure 2 shows a screenshot of the current prototype of the “toxicity searcher”. We believe that our search engine can be a valuable tool for nanomedical researchers to easily discover toxic and secondary effects of nanoparticles reported in the literature.

To our knowledge, the results we report are the first application of text mining methods to extract nanotoxicology information from the literature—in fact, the first text mining application in the whole field of nanomedicine. Considerable interaction between nanoinformatics professionals should enable building extended corpora in this and other fields, where challenges and competitive testing can be carried out to evaluate these methods from text mining, information retrieval, and how they perform with different information types. Similar competitions have been earlier carried out in BMI, with significant results and success [52, 54, 55]. In this way, we can consider our research as a first “proof of concept”, which needs to be followed up soon by efforts by others, and may provide opportunities in an entirely new area of research for nanoinformaticians.

Extending the research presented in this work to include more general entities—that is, nanomedicine and nanotechnology-related entities—can open new and significant challenges for nanoinformaticians, given the novelty of this topic and approach. These potential challenges include, for instance: (a) populating electronic health records and/or clinical trials with nanolevel information extracted from the literature, (b) automatically annotating and indexing nanomedical documents mentioning concepts and entities belonging to well-known ontologies and controlled vocabularies, (c) aligning and bringing together existing biomedical and nanomedicine/nanotechnology ontologies—such as for example the Nanoparticle Ontology [59], the Foundational Model of Anatomy [58], or the Gene Ontology [61]—or (d) automatically creating inventories of nanoparticles containing details about their characterization and design, potential uses, and applications—for example tissue regeneration, drug delivery, medical imaging, identification of cancerous cells, for example—toxicity, links to relevant literature, links to modeling, and simulation tools, and so forth. 

This research is an example of the potential challenges and synergies that lie ahead for future interactions between experts in nanotechnology, nanomedicine, and nanoinformatics. Such interactions may lead to a broad range of medical applications involving different nanomedical challenges. In this regard, the authors are currently working together on the development of new methods and tools for addressing these issues.

## 5. Conclusion

In this paper, we have presented a nanoinformatics approach based on NER techniques for automatically identifying relevant nanotoxicology entities in scientific articles. The results of the evaluation suggest that the entity recognizer, we have developed could be used by other nanoinformaticians to reliably perform different NER-dependant tasks. These include extracting nanotoxicity information from textual sources to populate structured databases, or to automatically index and search nanotoxicology articles. In addition, this work can be extended to recognizing more general nanomedicine and nanotechnology entities, thus providing new research opportunities for nanoinformaticians. This is, to our knowledge, the first report that explores the use of text mining techniques in the area of nanotechnology. Further research in this emerging nanoinformatics field may lead to the development of novel methods and tools that could assist researchers in managing data, information, and knowledge at the nanolevel, thus accelerating research in nanoscience. 

## Figures and Tables

**Table 1:** Number of entities and tokens manually identified by the annotators in the 300 selected phrases and annotated as belonging to one of the target categories.

	Nano	Expo	Toxic	Target	Total
Entities	426	144	485	385	1440
Tokens	717	186	637	705	2245

**Table 2:** Summary of results of the evaluation of the CRF-based recognizer using 10-fold cross-validation.

	Entity-level			Token-level		
	Precision (EP)	Recall (ER)	*F*-measure (FR)	Precision (TP)	Recall (TR)	*F*-measure (TF)
Nano	0.892	0.873	0.883	0.945	0.943	0.944
Expo	0.930	0.826	0.875	0.981	0.855	0.914
Toxic	0.926	0.874	0.899	0.967	0.909	0.937
Target	0.876	0.860	0.868	0.906	0.916	0.911

**Table 3:** Summary of results of the evaluation of the hybrid approach used as baseline.

Entity-level			
	Precision	Recall	*F*-measure
Nano	1.00	0.33	0.496
Target	0.75	0.48	0.585
